# *Taraxacum officinale* Seed Extract Inhibits HeLa Cell Migration at Sub-cytotoxic Concentrations

**DOI:** 10.17912/micropub.biology.001900

**Published:** 2026-01-13

**Authors:** Christina Hendrickson, Melville B. Vaughan, John Nail, Jeremy Dry

**Affiliations:** 1 Biology, Oklahoma City University, Oklahoma City, Oklahoma, United States; 2 Biology, University of Central Oklahoma, Edmond, Oklahoma, United States; 3 Chemistry , Oklahoma City University, Oklahoma City, Oklahoma, United States

## Abstract

Dandelion seed extract exhibits potent anticancer activity in HeLa cells. HeLa viability decreased with increasing doses of DSE from 0 to 0.8 mg/mL, while HDF viability was not affected under similar conditions. DSE reduced HeLa cells’ migration speed between 0-0.050 mg/mL, while no significant effect was observed in HDF cells over the same range. Both cell lines exhibited a significant decrease in cell migration speed at 0.1 mg/mL. This data suggests that DSE treatment inhibits cell migration before it significantly reduces cell viability. It points to a specific anti-migratory effect independent of viability loss.

**Figure 1. Dandelion Seed Extract’s Effect on HeLa and HDF Cell Viability:**
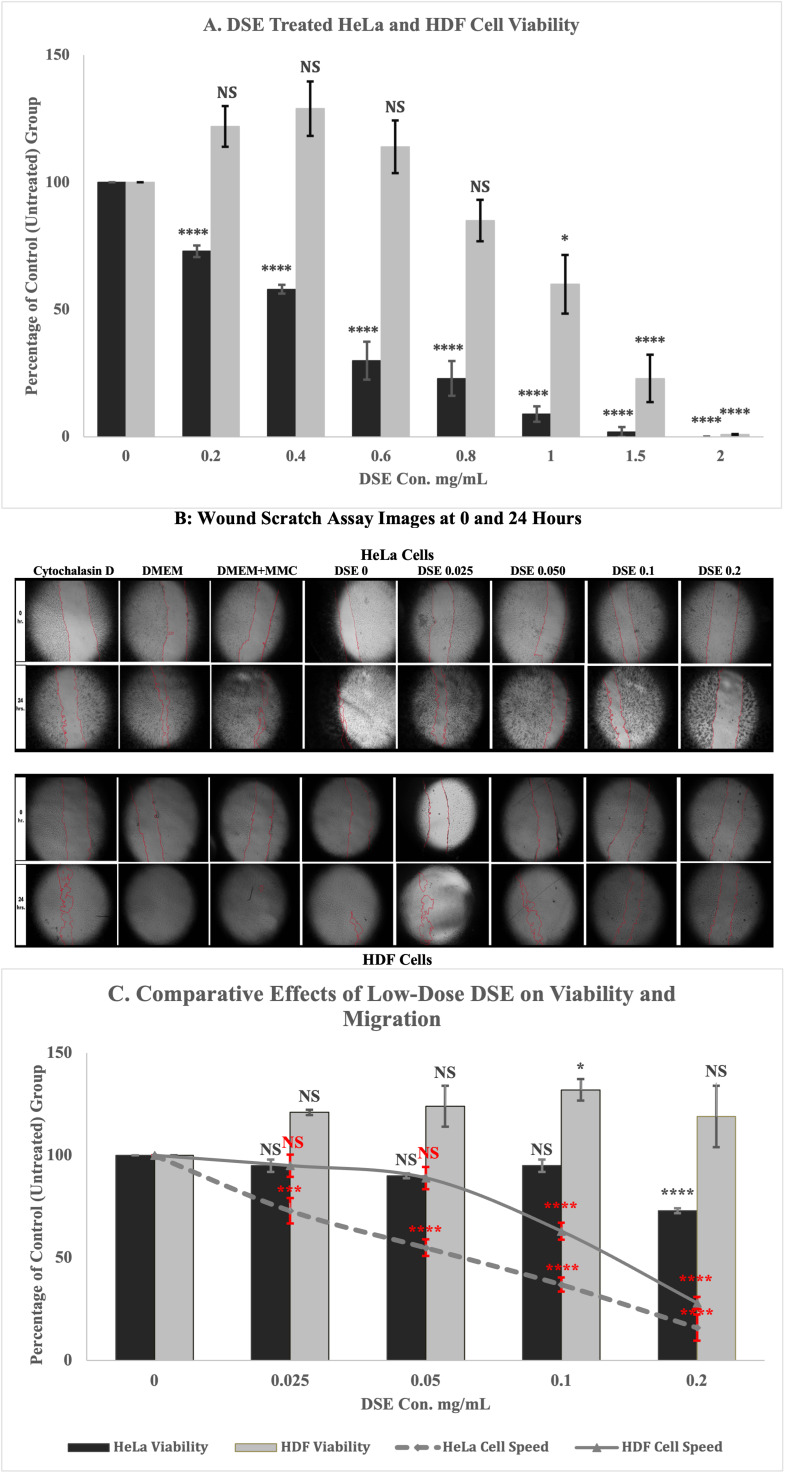
*A. The DSE effect on HeLa and HDF cell viability is determined by the PrestoBlue HS assay. The black bars represent HeLa cells, and the gray bars represent HDF cells* . *The percentage of cell viability after 24 hours was calculated in each treatment group based on 100% representing the vehicle control group (DMEM/10% FBS/2% DMSO). Statistical significance is indicated as follows:***** p > 0.05 (ns), p < 0.05 (*), p < 0.0001 (****); n = 12. B. DSE inhibits HeLa and HDF migration independent of proliferation. A confluent lawn of cells was scraped, washed, and treated as shown in the legend. Mitomycin C (MMC) treatment to reduce proliferation demonstrated similar migration compared to control, while Cytochalasin D pretreatment demonstrated delayed migration recovery after washout. n=9 C. DSE differentially affects viability and migration. Data for viability and migration were quantified and superimposed on a graph. Results indicate that migration effects were observed beginning at lower doses than those seen with viability effects. p > 0.05 (ns), p < 0.05 (*), p < 0.001 (***), p < 0.0001 (****).

## Description

Dandelion ( *Taraxacum officinale* ), commonly perceived as a pervasive weed, has attracted growing scientific attention due to its promising therapeutic properties (Scaria et al., 2020). Belonging to the Asteraceae family, *T. officinale* is widely distributed across the globe and has been utilized in traditional medicinal systems for centuries (González-Castejón et al., 2012).

Studies reveal that dandelion extracts possess anticancer properties. Dandelion root and seed extracts have demonstrated inhibitory effects on the proliferation and migration of multiple cancer types, including esophageal squamous cell carcinoma (Li et al., 2022), gastric cancer (Zhu et al., 2017), colorectal cancer (Ovadje et al., 2016), and melanoma (Chatterjee et al., 2011).

This study focused on evaluating the anticancer potential of *T. officinale* seed extract in HeLa cells, a human cervical carcinoma–derived cell line widely used as an *in vitro* model of cervical cancer (Higareda-Almaraz et al., 2013). Cervical cancer remains one of the most common and lethal cancers among women globally. In 2022, there were approximately 661,021 new cases and 348,189 deaths due to cervical cancer globally (Bray et al., 2024; WHO, 2024). Since the mid-1970s, cervical cancer incidence has declined by over 50% due to widespread HPV vaccination. However, rates have recently increased by 11% among women aged 30–44 between 2013 and 2021 (Siegel et al., 2025). The investigation of novel, low-toxicity, plant-derived compounds such as Dandelion Seed Extract (DSE) may offer promising avenues for adjunctive or alternative cancer therapies.

In our study, DSE inhibited the viability of HeLa cells in a dose-dependent manner, with less pronounced effects on HDF cells (Fig. 1). Cell viability measured 24 hours post-treatment demonstrated that HeLa cell viability decreased with increasing doses of DSE from 0 to 0.8 mg/mL, while HDF cell viability was not affected, compared to vehicle control, under similar conditions. Both cell lines exhibited a significant decrease in viability at higher concentrations of 1, 1.5, and 2 mg/mL. Statistical analysis confirmed a significant decrease in HeLa cells' viability upon increasing concentrations of DSE. Two-way ANOVA revealed a highly significant difference between the two cell lines ( *p =* 4.68 × 10⁻²⁷), indicating a strong overall effect of cell type on the outcome (partial η² = 0.484). Treatment concentration also had a highly significant impact ( *p =* 8.73 × 10⁻⁴⁸) with a large effect size (partial η² = 0.737). There was a significant interaction between cell line and treatment concentration ( *p* = 4.14 × 10⁻¹²), with a moderate to large effect size (partial η² = 0.316), indicating that the effect of DSE concentration on cell viability depends on the cell type. Specifically, increasing DSE concentrations elicited different viability responses in HeLa cells compared to HDF cells. Tukey HSD/Kramer post hoc analysis was performed following one-way ANOVA to determine pairwise differences among DSE treatment groups in each cell line. In HDF cells, DSE treatment did not significantly reduce viability at concentrations ranging from 0 to 0.8 mg/mL. However, a statistically significant reduction in viability was observed at higher concentrations of 1, 1.5, and 2 mg/mL with *p-values* of 0.026, 1.45×10⁻⁷, and 2.82×10⁻¹¹, respectively. In contrast, HeLa cells exhibited a highly significant reduction in viability at all tested concentrations, with *p-values* for the seven treatment groups as follows: 0.2 mg/mL ( *p* = 9.29 × 10⁻⁵), 0.4 mg/mL ( *p* = 1.23 × 10⁻⁹), and 0.6–2 mg/mL ( *p* = 8.99 × 10⁻¹⁴). These results indicate a dose-dependent cytotoxic effect of DSE in HeLa cells, in contrast to the limited sensitivity observed in HDF cells ( Fig. 1A ).

The effect of DSE on cell migration was tested by scratch wound assay. HeLa cells and HDF cells were subjected to a wound scratch assay and treated with DSE concentrations of 0-0.2 mg/mL. DSE reduced HeLa cells’ migration speed (µm/hr) in a dose-dependent manner between 0–0.050 mg/mL, while no significant cell speed reduction was observed in HDF cells over the same range. However, both cell lines exhibited a significant decrease in cell migration speed at 0.1 mg/mL ( Fig. 1B and C).

The viability and migration data, when combined, showed that DSE began to affect migration at lower doses than observed with viability ( Fig. 1C ). This data suggests that DSE treatment inhibits cell migration before it significantly reduces cell viability. It could point to a specific anti-migratory effect independent of viability loss. For statistical analysis of cell migration, a two-way multivariate analysis of variance (MANOVA) was performed using Pillai’s Trace to assess the effects of cell line and DSE dose on cell viability and migration speed. The analysis revealed significant main effects for the cell line (F = 93.01408, *p* = 1.53×10⁻¹⁰) and DSE dose (F = 11.96301, *p* = 1.62×10⁻⁸), as well as significant interaction between the two factors (F = 3.465426, *p* = 0.004). Although the test was labeled as Pillai’s Trace by the Real Statistics Using Excel, the trace statistic itself was not reported. Partial eta-squared values indicated substantial effect sizes for each factor (η²ₚ for cell line = 0.9, for DSE dose = 0.7, and for cell viability and migration = 0.4).

The anticancer effects of DSE observed in this study appear to result from a combination of mechanisms targeting both cell migration and proliferation. In DSE-treated HeLa cells, a primary mechanism may involve the disruption of signaling pathways associated with migration and metastasis. By interfering with transduction pathways that regulate cell growth and proliferation, DSE impairs critical processes such as cell cycle progression, mitotic entry, and cytoskeletal organization, ultimately contributing to tumor growth inhibition. Functional assays demonstrated that DSE suppresses HeLa cell migration at lower concentrations, while cytotoxic effects on cell viability become more pronounced at higher doses. These findings suggest a dose-dependent, multi-stage mechanism in which DSE initially reduces metastatic potential before inducing cytotoxicity in cervical cancer cells. In contrast, normal HDF cells maintained higher viability and cell migration, supporting the cancer-selective action of DSE. These findings position DSE as a promising candidate for further exploration as a targeted therapeutic agent. Future studies are warranted to evaluate its efficacy in additional cancer models, as well as its safety and potential vascular effects in endothelial systems.

## Methods

**Dandelion Seed Extract Preparation**

*Taraxacum officinale* seeds (indeed achenes) were purchased from Seed Needs LLC, New Haven, MI, USA, via Amazon, whose species was confirmed as *Taraxacum officinale* by Oklahoma City University (OCU) botanist Adam Ryburn, Ph.D. Dandelion seeds were extracted using a Soxhlet apparatus for at least 12 hours with a mixture containing 5% (v/v) 2-propanol in ethanol, which had been denatured with both 2-propanol and methanol. Volatiles were removed by rotary evaporation to produce a greenish solid. This solid was dissolved in dimethyl sulfoxide (DMSO) to yield a concentrated dandelion seed extract of 100 mg/mL.

All extract batches were prepared from the same commercially sourced seed lot using identical extraction materials and conditions. To ensure consistency between independently prepared batches, the biological activity of each DSE preparation was assessed by cell viability assays prior to and during experimental use. Comparable dose-dependent effects were observed across batches. The anticancer activity of DSE was evaluated using HeLa cells and human dermal fibroblasts (HDF).

**Cell Cultivation**

HeLa cells (ATCC® CCL-2™) purchased from American Type Culture Collection (ATCC), Manassas, VA, USA, and HDF cells (FC-0024, lot number 1035) purchased from LifeLine Cell Technology, Frederick, MD, USA, were selected as model cell lines for this study. HeLa and HDF cells were cultured in Dulbecco’s Modified Eagle Medium **(** DMEM, high glucose, Gibco) with 10% fetal bovine serum (Biowest LLC, catalog no. S-1620) and 1% antibiotic-antimycotic (Sigma, A5955). Cell cultures were maintained at 37°C, 5% CO₂, and 90% humidity.

**PrestoBlue HS Cell Viability Assay**

Cell viability was assessed using PrestoBlue™ HS Cell Viability Reagent (Thermo Fisher Scientific, Cat #P50200), following the manufacturer’s protocol (Thermo Fisher Scientific, n.d.). Experiments were conducted using two reagent lots (Lot #2906449 and Lot #3140593), which produced consistent results across replicates. To conduct PrestoBlue HS, 10 μL of reagent was added to the wells of the 96-well plate (Thermo Scientific™ Nunc™ MicroWell™) containing 2 x 10 ^4^ cells treated with varying concentrations of DSE (0-2 mg/mL) for 24 hours and then incubated for 10 minutes at 37℃, 5% CO₂, and 90% humidity. The conversion of resazurin to fluorescent resorufin was quantified using a Synergy H1 microplate reader (BioTek, Winooski, VT, USA) at an excitation wavelength of 560 nm and an emission wavelength of 590 nm. The collected data was analyzed by Microsoft Excel. The cell viability percentage for each treatment was calculated using the vehicle control as the baseline.

**Cell Migration Assay**

A wound scratch assay was performed to assess DSE-treated and non-treated HeLa and HDF cells. 4 x 10 ^4^ cells were seeded in triplicate in each well of 96-well plates (Thermo Scientific™ Nunc™ MicroWell™). At 100% confluence and prior to scratching, cells were pretreated with 5 µg/mL MMC (Mitomycin C) in 2% serum-containing medium for two hours to inhibit cell proliferation during the migration assay. As a migration-inhibited control, a separate group of wells was pretreated with 1 µM Cytochalasin D, an actin polymerization inhibitor known to block cell motility (Justus et al., 2014). After three washes, a uniform scratch was made in each well using a sterile 200 µL pipette tip. The wells were then washed again to remove cell debris and detached cells. Scraped wells were treated with different concentrations of DSE, from 0 to 0.2 mg/mL.

Cell migration was photographed immediately after treatment and after 24 hours using an Amscope inverted light microscope with a MU300-HS camera. The distance of wound healing closure was measured by ImageJ software at least 8 times in each replicate. Measurements were subjected to further analyses using Excel to evaluate cell speed (µm/hr) following DSE treatment.
